# Genoa

**Published:** 1888-03

**Authors:** A. Lagorio

**Affiliations:** Genoa, Italy


### FOREIGN CORRESPONDENCE.

Mr. Editor: It is a fact that in Italy as well as in Germany, some of the best schools of medicine are not to be found in the large cities. Our ancestors probably were right in thinking that tranquility was a necessary thing to students desirous to penetrate the regions of science, and that their minds could better be concentrated upon their favorite studies in small centres, where distractions and enticements incident to life were to be far less found. This is especially applicable to Pavia, which, although being only a secondary city, possesses the most honored university of the kingdom. Milan, the capital of Lombardy, distant only one hour by train from Pavia, has a population of over 300,000 inhabitants, a magnificent hospital, and other sanitary institutions, and yet has no university. Padua, Pisa, etc., are only second and third rate cities, but their universities are world renowned.

My recent trip to Pavia gave me an opportunity to visit her great university, and my gratification was such that I concluded to send you this communication.

The city of Pavia has at present 35,000 inhabitants. Obscure as is her origin, it is said that it dates back many centuries before Christ; and, although now of not much importance, yet once she was the capital of an important republic, and the home of many emperors. The city itself cannot be called beautiful, but is situated upon the most fertile field of Lombardy, and is widely known for her celebrated university.

Records cannot be found to enlighten us on the origin of the university, but it is known that even before the ninth century the university of Pavia enjoyed a foremost reputation, and in the year 1361 it became officially recognized by a diploma of Charles VI granting the right to teach law, philosophy, medicine, and arts.

The present building is a large one, two stories high, with a frontage 185 metres long. It is divided inside by five large open court-yards, surrounded by five porticoes. Under the colonnades there are several inscriptions and monuments dedicated to some of the past illustrious teachers. On the first floor are situated the anatomo-pathological museum, the museum of normal anatomy, and the anatomical theatre. The latter is a well-lighted large hall, artistically painted, and was inaugurated by Scarpa in the year 1785. It contains an admirable bust of this great anatomist. Wide stairs lead us to the second story, similar in all particulars to the lower one. It is also ornamented with a fine internal colonnade, busts, and inscriptions. On this floor are situated the zoological museum, as well as that of comparative anatomy, of geology, and of mineralogy, and an excellent anatomo-pathological laboratory. Several lecture-rooms are situated here and there; the most admirable of these is that of physics. It contains the life-size statues of Galileus and of Cavalieri; also two bass-reliefs representing Europe with Newton's effigy, and America with Franklin's effigy. In this room Volta delivered his famous lectures, and a bust of him is placed on the wall in the centre of the hall, and bears underneath the following epigraph:

*Alexander Volta*

*in re electrica princeps*

*vim raiæ torpedinis meditatus*

*naturæ interpres et æmulus.*

Noteworthy in this university is the botanical institute. The laboratory is furnished with over thirty microscopes and several microtomes; has a rich collection of specimens of the vegetable anatomy and pathology; also several chemical and physiological apparatuses. The garden is supplied with about fifty thousand plants, and the library with over five thousand volumes.

The museum of zoology had its origin in the year 1775, in a rich collection donated by Spallanzani; in 1786 the Van-Hocq collection was added, and ever since it has been constantly amplified to its present dimension. Many rare animals can be seen in this museum, and most characteristic is the collection of the lacustral pelagic fauna. All the genera and species are arranged as in the catalogues of the British Museum, and as Claus' classification.

The first specimens in the museum of comparative anatomy were prepared by the celebrated Scarpa (1782). A rich collection has been added ever since by his followers. The museum contains a fine collection of skeletons; also many specimens of the nervous system of the cuttle-fish and torpedo prepared by Scarpa; many embryological wax preparations and numerous animal monstrosities.

The institute of normal anatomy has great traditions. It is probably sufficient to name Carcano, Aselli, Scarpa, Porta, who have so much honored this institute and made it foremost in Italy as well as abroad. The number of preparations collected in the museum is 2,387, and are placed in four large halls. In the department of osteology there can be seen a large collection of skeletons of all ages (from 101 years down to two months of gestation); a collection of skulls of individuals of different races, or of a different anthropological type; a group of skulls of famous men, as of Scarpa, Volta, and others, who stood high in all sciences. In the department of angiology I saw four complete human statues and several special preparations; a grand collection of lymphatic injections made with mercury by Panizza. Notable are also two wax statues showing all the lymphatics of the body. Noteworthy are the historical preparations of Scarpa of the olfactory nerves, those of Panizza of the nerves of taste, and a large wax model of the organ of hearing, which received the prize at the London exhibition of 1862. In the department of embryology there were to be seen several injections of the fœtal and maternal cotyledons prepared also by Panizza. There was also an Egyptian mummy and the body of a man whose skin was as mottled with pigmented maculæ as the skin of a leopard. The theoretical teaching of anatomy in this university is triennial.

Experimental physiology has been taught since 1783, by Rezia, Jacopi, Hildebrand and others. The laboratory is well furnished with a complete armamentarium for vivisections, with several microscopes, electric batteries, saccharometers, spectroscopes, cardiometers, pneumathometers, etc., etc. Notable is also the collection of anatomical and histological preparations. The teaching in this branch is biennial.

The institute of general pathology and histology was founded in 1863 by Montegazza. Professor Bizzozero followed in in 1869, and since 1879 it has been under the direction of Professor Golgi. In 1884 there was added a laboratory for bacteriological researches, furnished with thirteen splendid microscopes, microtomes, etc.

The library of the university is worthy of special attention. Since 1754 it was recognized that a library was much needed, but it was not until 1772 that it was founded by Fontana. Haller's library was soon added to it. The celebrated Joseph Frank also bequeathed a large sum, and directed that the interest derived from it be employed to buy works on medicine and natural sciences, and that these be added to the library. Since then the library has been increased to the present large dimensions and importance by many donations from the government, citizens, and scientific institutions. The number of volumes is 120,000, with the addition of 70,000 pamphlets. Notable is the collection of historical works. Worthy of note are the anatomical tables of Bourgery, Cruveilhier, Blainville, Barkow, and those of Hebra on skin diseases; Mondino's Anatomy, edited in 1478; the medical works of Falcucci, published in 1481–84; the principal edition of Celsus' works, brought to light at Florence in 1478, and the little work of Cananus on the muscles of the human body, which is thought to have been printed about the year 1541, and of which only four copies are believed to be in existence.

The teaching of materia medica was commenced in 1770, by Professor Borsieri, who at that time had to lecture on chemistry, pharmacy, and clinical medicine. The laboratory occupies five rooms, and the museum is supplied with a rich collection of medicinal substances, especially of exotic plants.

The museum of morbid anatomy occupies three rooms, and has about 1,600 specimens. In the laboratory are placed the specimens of pathological histology, and the reports of 7,614 autopsies. The teaching in this department is biennial.

Michel Rosa first taught hygiene in 1767. Among the illustrious teachers in this department mention must be made of G. Peter Frank, who wrote the first large treatise on hygiene.

The laboratories of medical jurisprudence, of general and pharmaceutical chemistry, are well provided with instruments, etc.

By looking over the history of the university I found that even in the fifteenth century Pavia was well supplied with eminent teachers. Among these I will mention A. Guaineri, who taught medicine in 1455, and Ferrari Dagrado, who read medicine and philosophy from 1432 to '72.

In the beginning of the sixteenth century the studies were interfered with by many wars, but these having ceased, the university soon regained her former glory. Of the teachers, I find the names of G. Cardano (1536), a most learned encyclopædian, who left many valuable works on medicine and mathematics. J. B. Leo Carcanus (1573), a reputed anatomist, whose observations, especially on the heart, we hear frequently spoken of in medical literature. Over three hundred auditors used to attend his lectures.

In the latter half of the sixteenth century horrible wars again broke out, bringing with them poverty and pestilence. A period of decadence then began for the university; all her former privileges were withdrawn, and the number of students diminished considerably. Of the teachers who upheld the old noble traditions of the Pavesian school, we must remember the modest Gaspar Aselli, the discoverer of the lacteal vessels, who taught anatomy in the year 1624. His discovery dates anterior to that of Harvey's on the circulation.

During, and since the eighteenth century, the university gained in honors and students, and had as teachers men who stood high in science and letters. Among these:

L. Spallanzani, called to the chair of natural history in the year 1769. His studies and experiments marked a new era in physiology. By numerous experiments on artificial fecundations he cleared the way for an understanding of the fundamental laws of generation in vegetables as in animals. He discovered the white blood-corpuscles in the salamander and in frogs; and was the first to study artificial digestion. Besides all these, he made many important researches, in the groundless belief of a sixth sense of the bats, on the respiration, circulation, on the phosphorescence of the sea, and on the volcanic phenomena; all of which greatly increased the patrimony of science.

G. A. Scopoli held with honor the chair of chemistry and botany from 1776 to 1788. He studied the metals, the fossils, and the insects. Many plants, which he classified, bear yet the same name given by him.

L. V. Brugnatelli followed Scopoli in 1788. He discovered the suberic acid, the erythric acid, the fulminating silver; he also deserves the credit of discovering uric acid in the excrements of the silkworm; and we owe to him the first observations on galvanoplastic. He wrote a reputed treatise on chemistry and materia medica, and honor is due him for the great zeal with which he promoted the diffusion of chemical studies in Italy and abroad.

Alexander Volta, who had already become famous for the discovery of the electrophorus and for his researches on the electrical capacity and on the inflammable gas, was elected professor at Pavia in 1778. Here he invented the electric condenser, made numerous studies on the atmospherical electricity, and in January, 1800, he constructed the pile. This great man, worthy of the highest place in the history of civilization, left the chair in 1813, and died in 1827. A life-size marble statue of him can be seen in the centre of the western court-yard.

Peter Moscati held the chair of anatomy from 1764 to 1772, and improved with much zeal the anatomical studies which had been much neglected. He held that there was no substantial difference between the inferior animals and man, and that the primitive posture of man ought to have been that of a quadruped. He was made a count and a senator by Napoleon I, and received many special favors.

Antonio Scarpa was called to Pavia in 1783. The university owes to Scarpa the celebrity of its anatomical and surgical school. Here he made his discoveries on the organ of hearing; he described minutely the olfactory nerves and the anterior nasal nerves; he illustrated the topographical anatomy of the limbs, teaching the rules for the ligatures of the arteries; he studied the diseases of the eyes, demonstrating how the clinical observation must be based on the anatomy; and lastly wrote two works on hernia and on aneurism which have remained classic.

S. A. Tissot taught practice of medicine in 1781. He wrote important works for those times, on nervous diseases, on onanism and sexual diseases, which made his name popular.

G. Peter Frank succeeded Tissot in 1785, and was much honored for his learning.

Joseph Frank took his father's place in 1795. He followed for a time Brown's theories, but soon abandoned them.

V. L. Brera followed. He diffused vaccination, and wrote a grand work on worms and their diseases, which was translated into all the principal languages.

In the year 1797 G. Rasori was called to the chair of clinical medicine. Forgetting that the observation of facts is the only basis of science, he permitted himself to be deluded by the simplicity of philosophical speculations and founded the famous "contra-stimulant" method, which consisted in bleeding and the use of tartar-emetic. His teaching was bitterly fought, so much so that after a year he was obliged to leave the chair.

Vincenzo Malacarne, already famous for his surgical works and for his excellent anatomical observations on the nervous centres of man and animals, was elected professor of surgery and obstetrics in the year 1789. He wrote a much honored treatise on "Exploration as a fundament in the obstetrical art."

Bartolomew Panizza, Scarpa's pupil, was elected professor of anatomy in 1815. He paid much attention to ophthalmology, and published a valuable essay "On the medullary fungus of the eye and on the depression of cataract." He gave an original description of the lymphatics of the genitals, demonstrated with mercuric injections. He also showed the absence of the vessels in the skin, and confuted the pretended Lippi's discovery, who taught that the lymphatics opened in veins. Later, he accurately described the lymphatic system of reptiles, and made known the inter-aortic opening of crocodiles, named by Brücke *Foramen Panizzæ*. In his experimental researches on nerves he discovered the function of the spinal roots, and demonstrated the functions of the glosso-pharyngeal nerve. Worthy of attention are his teratological observations, as well as his researches on the marine lamprey, in which he found the sexual demorphism and many other anatomical facts; also his experimental researches on the optic nerve; and many other studies, as on the gravid uterus. In 1860 he was elected senator; in 1864 he left the chair, and died in 1867. To perpetuate the memory of such a worthy teacher a fine statue was erected to him in 1873, and it can be seen under the internal colonnade of the university; but a far more durable monument was built by Panizza himself in his works.

Mauro Rusconi's name will also forever be known for his great discovery, in 1826, of the segmentation of the ovum, and other important studies on embryology.

Frank Flarer taught ophthalmology in Pavia from 1819 to 1859. Hirsch names him as a worthy representative of the Vienna school in Italy.

F. S. Hilderbrand taught clinical medicine from 1817 to 1830, and studied the means to combat the pellagra.

T. Lovati held the chair of obstetrics from 1827 to 1848. He introduced in Italy the artificial premature labor, and was the first one to use auscultation in obstetrics.

Luizi Porta held the chair of surgery for forty years. His reputation as a surgeon and an anatomist was, and is yet to-day, widespread throughout the world. His memory is also perpetuated with a beautiful statue placed under the colonnade. He bequeathed all of his large fortune to the university fund, besides a fine collection of 260 preparations of surgical anatomy, and over 1,400 preparations of surgical pathology. All these are placed in three large rooms under the name of Porta's Museum.

Among the living who have made important studies at this university, and have held chairs, adding much glory to this already famous atheneum, the following are worthy of special mention :

Salvator Tommasi, who taught clinical medicine from 1859 to 1864. He held constantly in his lectures that the duty of the physician is to study the sick man as a physiologist studies a sound organism ; that disease is not a new being with different laws governing the organism, but only a deviation in the typical progress of the organic laws. He is at present at Naples, much honored and admired.

Paul Mantegazza taught experimental pathology from 1860 to '69. He did in this institution much of his scientific work on the spermatic fluid, on the genesis of fibrine, on the persistency of vibriones in boiled liquids, on animal inoculations, on the temperature of urines, etc. He has been, and is to-day, a prolific writer. His writings on hygiene, travels, and many physiological and philosophical works, are universally read and admired.

C. Lombraso taught psychiatrics from 1864 to '76. He advocated with energy the necessity of the study of anatomy in mental diseases, and was the founder of criminal anthropology. He is now in Turin.

The name of G. Bizzozero is much esteemed at home as well as abroad. He taught general pathology from 1869 to '72, and during that time he completed a series of studies on the structure of the pineal glands, on the marrow of bones, on the histology of epithelia, etc. He now holds the same chair in Turin, where he continues his observations with much conscientiousness. His last work on clinical microscopy is generally read, and has been translated in all the European languages.

Who doesn't know or at least has not heard of Arnaldo Cantani? This illustrious clinician commenced his scientific career at this university, and he did much to found and diffuse in Italy the scientific method in the clinique. He is now connected with the University of Naples, where he holds the highest place as a teacher and clinician.

E. Porro taught obstetrics for many years. Here he performed the classic operation of utero-ovarian amputation which bears his name. He has retired to private practice in Milan.

All the clinical teaching at this university is imparted at St. Matthew's Hospital. This hospital was built in the year 1449, hence it has many defects consequent upon the manner of building in those old times; but in these later times many additions have been made, and the old part has been remodeled to coincide with all the requirements of the modern progress of hygiene. The hospital is sustained by revenues of trust funds which reach the sum of nearly a million of francs. The hospital has a fair library, due to the generosity of Professor A. Brambilla, who also donated a complete armamentarium of surgical instruments. In these later years the medium mortality has been ten per centum in the department of medicine and six per centum in that of surgery. The diseases which have made the mortality so much in surgery are mostly gangrenous erysipelas, inflammations and vast suppurations.

Recently there has been instituted in this hospital a school for nurses with good effect. The teaching consists in the elements of anatomy, physiology, surgical help, the manner of assisting patients, dietetic régime, some idea of diseases, on hygiene, etc.

The medical clinique has been in charge of Professor F. Orsi, since 1865. The fifth and sixth year students only attend this clinique, which is situated on the ground-floor of the hospital, and is divided into two large rooms, each holding thirty beds.

The surgical clinique was, until last September, in charge of Professor Henry Bottini, who gave up his clinique, having been elected a member of the Italian parliament, a fact much regretted by the profession which loses a most eminent clinician and surgeon. All the operations are performed in the surgical amphitheater of the hospital, which is adorned with inscriptions, two marble busts of Scarpa and Brambilla, and a beautiful collection of instruments; of these are worthy of mention those for operations on the urinary passages, those for the galvano-caustic operations, much improved by Bottini, those for diseases of the osseous system, and a most complete armamentarium for hemostasis. Over two hundred capital operations are here performed every year, such as removal of goitres, sarcomata, lymphomata, amputation of the tongue by a special method, resections, etc. This clinique is also provided with ample laboratories for histological and bacteriological researches.

There are several other cliniques here, but time and space do not permit me to give any detailed account of them. As the reader will see, this university has a splendid history, and its renown will increase in the future. Here, as in other Italian institutions, much scientific work is done, which, if this fact were made known, as is done in other nations, would divert in in this direction not a little of the travel of medical students who go abroad for advanced professional instruction. But the time will come when students and physicians will avail themselves of the fine opportunities offered in this beautiful sunny land, where, besides the excellent clinical instruction, the sojourn will be made far more profitable by the pleasant surroundings, charming climate, arts, music, language, and the courtesy which so much characterizes the Italian nation.

The corner-stone of a new Polyclinic was laid on the 19th of January at Rome. The King performed the act in the presence of the Queen, the Prince-heir, and all the prominent officials of the capital. Professor G. Baccelli, the originator of this new institution, made a brilliant address. Honor is due him for having conceived the idea to harmonize school and recovery, learning and philanthropy; and to place at the disposal of medical science a union of several clinics, to benefit science while benefiting charity. This institution will be erected upon the plans of architect G. Podesti; and the area of ground on which this great work is to be situated is just outside of the celebrated Porta Pia, where, eighteen years ago, the Italian army, after a sharp resistance, made a grand entrance into the new capital. The area is surrounded by boulevards thirty metres wide, and which measure 160,000 square metres.

The main building, in which are to be located all the offices, store-rooms, warden's residence, etc., is situated in the centre and in front. From this there will extend several galleries by which it will communicate with the other buildings. The main frontage will measure 560 metres long, and 300 metres deep. In all there will be over forty pavilions. A wide underground gallery will be used for the removal of cadavers to the dead-house, and also of all the clothes to the laundry. Above this gallery there will be a portico to communicate with all the infirmaries. The medical and surgical clinical wards, with the annexed hospital, will be situated on the side of the main building. Special pavilions for infectious and contagious diseases, as well as the dead-house and laundry-rooms, will be built in the corner farthest from the centre of the area. These will be reached only by underground galleries, with a view of avoiding infection. The balance of the ground will be changed into a beautiful garden.

It is intended to make it, when erected, the largest and the best equipped institution devoted to medical teaching in Italy. It is to be hoped that it will meet all the requirements of modern sanitary science, and, as the first one of the kind, be an honor to Rome and Italy, and thus be a glorious page added to the history of the Eternal City. A. LAGORIO, M. D.

GENOA, ITALY, February 7, 1888.

